# Assembly of the Yeast Exoribonuclease Rrp6 with Its Associated Cofactor Rrp47 Occurs in the Nucleus and Is Critical for the Controlled Expression of Rrp47*

**DOI:** 10.1074/jbc.M112.445759

**Published:** 2013-04-11

**Authors:** Monika Feigenbutz, Rebecca Jones, Tabot M. D. Besong, Stephen E. Harding, Phil Mitchell

**Affiliations:** From the ‡ Molecular Biology and Biotechnology Department, University of Sheffield, Firth Court, Western Bank, Sheffield S10 2TN, United Kingdom and; the § National Centre for Macromolecular Hydrodynamics, University of Nottingham, Sutton Bonington, Leicestershire LE12 5RD, United Kingdom

**Keywords:** Analytical Ultracentrifugation, Microscopic Imaging, Nuclear Translocation, Protein Cross-linking, Protein Degradation, Ribonuclease, RNA-binding Protein, Yeast Genetics

## Abstract

Rrp6 is a key catalytic subunit of the nuclear RNA exosome that plays a pivotal role in the processing, degradation, and quality control of a wide range of cellular RNAs. Here we report our findings on the assembly of the complex involving Rrp6 and its associated protein Rrp47, which is required for many Rrp6-mediated RNA processes. Recombinant Rrp47 is expressed as a non-globular homodimer. Analysis of the purified recombinant Rrp6·Rrp47 complex revealed a heterodimer, suggesting that Rrp47 undergoes a structural reconfiguration upon interaction with Rrp6. Studies using GFP fusion proteins show that Rrp6 and Rrp47 are localized to the yeast cell nucleus independently of one another. Consistent with this data, Rrp6, but not Rrp47, is found associated with the nuclear import adaptor protein Srp1. We show that the interaction with Rrp6 is critical for Rrp47 stability *in vivo*; in the absence of Rrp6, newly synthesized Rrp47 is rapidly degraded in a proteasome-dependent manner. These data resolve independent nuclear import routes for Rrp6 and Rrp47, reveal a structural reorganization of Rrp47 upon its interaction with Rrp6, and demonstrate a proteasome-dependent mechanism that efficiently suppresses the expression of Rrp47 in the absence of Rrp6.

## Teaser

**Background:** The Rrp6·Rrp47 complex is important for RNA processing and degradation events.

**Results:** Rrp6 and Rrp47 are independently imported into the nucleus, and Rrp47 is destabilized in cells lacking Rrp6.

**Conclusion:** Rrp6 binds Rrp47 in the nucleus and protects it from proteolysis.

**Significance:** Nuclear assembly of the Rrp6·Rrp47 complex spatially limits the nuclease complex and controls the expression of Rrp47.

## Introduction

The exosome complex is a major component of the ribonuclease armory of eukaryotic cells. The complex was initially identified in the budding yeast *Saccharomyces cerevisiae* through its role in the 3′ end maturation of small stable RNAs (1, 2) and subsequently shown to function in diverse nuclear and cytoplasmic RNA quality control mechanisms that target both stable RNAs and mRNA transcripts (3–5). Notably, genetic inhibition of exosome activity has led to the discovery of novel classes of transiently expressed RNAs (6–11) and demonstrated that a proportion of newly synthesized “stable” RNAs is in fact rapidly degraded (12–14).

Early biochemical fractionation and salt gradient elution experiments demonstrated that the yeast exosome has a minimal “core” structure, consisting of six subunits that show homology to bacterial RNase PH enzymes (Rrp41, Rrp42, Rrp43, Rrp45, Rrp46, and Mtr3) and three further proteins (Rrp4, Rrp40, and Csl4) that contain S1 and KH (K homology) RNA-binding domains (2). Consistent with these findings, all nine core subunits were required to reconstitute stable yeast and human exosome complexes *in vitro* (15). The core exosome complex from yeast and human cells is itself noncatalytic but is associated with the ribonucleases Rrp44 (also known as Dis3) and Rrp6. Rrp44 is related to the RNase II family of 3′ → 5′ exoribonucleases and also has an endonuclease activity associated with its N-terminal PIN (PilT N terminus) domain (16). Rrp44 and the core exosome are found in both the nucleus and the cytoplasm. In contrast, yeast Rrp6 is restricted to the nucleus (2, 17) and has often been used as a marker for nuclear exosome activity. Furthermore, interactions between the exosome and additional proteins, such as the TRAMP complex, the Nrd1·Nab3 termination complex, the Ski complex, or the RNA-binding proteins Rrp47 and Mpp6, are crucial for the processing or degradation of its diverse substrates (18).

Rrp6 belongs to the RNase D family of 3′ → 5′ exonucleases that is characterized by the presence of a “DEDD” catalytic domain, containing four conserved acidic residues (DEDD), followed by two HRDC (helicase and RNase D C-terminal) domains (19, 20). Rrp6 and its eukaryotic homologues are considerably larger than their prokaryotic RNase D counterparts and also have an extended C-terminal region of low structure complexity and, typically, an additional N-terminal PMC2NT domain (21). Conserved residues within the first HRDC domain are required for Rrp6 function (22), whereas the second C-terminal HRDC domain is required for the interaction between Rrp6 and the core exosome (19). The available partial structure of yeast Rrp6 suggests that the PMC2NT domain is wrapped around the catalytic domain (23).

Although Rrp6 is stably associated with the nuclear exosome complex in yeast cell lysates, core-independent roles for Rrp6 have been proposed in both yeast and flies (19, 24) and are supported by recent genome-wide analyses of exosome substrates in yeast (13, 14). In contrast to Rrp44 and the core exosome subunits, Rrp6 is not essential for viability, but the enzyme is required for optimal mitotic growth and *rrp6*Δ mutants exhibit a tight temperature-sensitive phenotype (25). Loss of Rrp6 expression or catalytic activity causes a dramatic accumulation of transiently expressed noncoding RNAs (6, 26), abortive RNA polymerase II transcripts (7), and stable RNA degradation intermediates (12) as well as delaying the 3′ maturation of small stable RNAs and eliciting an associated accumulation of 3′ end processing intermediates (25, 27–29). Many of the RNAs that accumulate in *rrp6* mutants are oligo- or polyadenylated. The TRAMP complex polyadenylates nuclear exosome substrates and stimulates their degradation (6, 30, 31).

The function of Rrp6 in pre-rRNA processing, 3′ maturation of small stable RNAs, degradation of cryptic unstable transcripts, and the surveillance of stable RNA production is also dependent upon the small basic protein Rrp47 (also known as Lrp1) (32–34). Rrp47 directly interacts with the N-terminal PMC2NT domain of Rrp6, and deletion of the PMC2NT domain elicits similar phenotypes to the loss of Rrp47 (35). Rrp47 has a conserved N-terminal Sas10/C1D domain (36, 37) that mediates the interaction with Rrp6 and that is critical for function of the protein, a variable C-terminal region that is predicted to be poorly structured and that is required for interaction with factors involved in the 3′ maturation of small nucleolar RNAs, and a highly basic C terminus that contributes to its *in vitro* RNA binding activity (38). Rrp47 is not required for the stable expression of Rrp6 or the association of Rrp6 with the exosome complex (32), but it is required for both exosome core-dependent and core-independent functions of Rrp6, such as the degradation of the 5′ external transcribed spacer fragment of the pre-rRNA and the 3′ maturation of 5.8 S rRNA and small nucleolar RNAs, respectively. This strongly suggests that assembly of the Rrp6·Rrp47 complex is an important step to ensure proper functional competence of Rrp6.

Little is known about the assembly pathways of exosome complexes or the spatial control of their constituent ribonuclease activities. Rrp6 is expressed in the nucleoplasm and nucleolus in yeast (2, 17), although there is a minor yet significant proportion of the enzyme found in the cytoplasm of cells from *Drosophila melanogaster*, *Arabidopsis thaliana*, *Trypanosoma brucei*, and humans (39–42). Yeast Rrp6 has a bipartite nuclear localization signal close to its C terminus (25), and a mutant Rrp6 protein lacking the nuclear localization signal is mislocalized to the cytoplasm (22), suggesting that its nuclear import is predominantly mediated via the importin-α/β heterodimer (Srp1 and Kap95 in yeast). Consistent with this, Srp1 and Kap95 are found associated with Rrp6 in pull-downs from yeast cell lysates (33, 43). Yeast Rrp47 and its human homologous protein, C1D, are also localized to the cell nucleus (44, 45). Intriguingly, some observations suggest that the interaction between Rrp6 and Rrp47 might be important for their subnuclear localization in human cells and in some plants. First, C1D is no longer localized to the nucleolus of human HEp-2 cells upon depletion of PM/Scl100 and instead accumulates in the nucleoplasm (46). Second, of the two nuclear Rrp6 proteins found in *A. thaliana*, Rrp6L1 lacks a PMC2NT domain and is localized to the nucleoplasm, whereas Rrp6L2 has an N-terminal PMC2NT domain and is targeted to the nucleolus (42).

Here we report our studies on the assembly of the Rrp6·Rrp47 complex in the yeast *S. cerevisiae*. Our findings reveal that assembly of the Rrp6·Rrp47 complex occurs primarily in the yeast cell nucleus after independent nuclear import of the two proteins and protects Rrp47 from proteolytic degradation. We suggest this serves both to spatially limit the expression of the assembled Rrp6·Rrp47 complex within the nucleus and to prevent inappropriate expression of Rrp47.

## EXPERIMENTAL PROCEDURES

### 

#### 

##### Strain Construction and Growth

Plasmids were generated by standard restriction digestion cloning or by site-directed mutagenesis using the QuikChange kit (Agilent Technologies). Constructs generated by PCR were verified by sequencing. Gene deletions and integrations were performed by homologous recombination using PCR-amplified DNA. Transformants were isolated on selective growth media and screened by PCR. Yeast strains were routinely grown at 30 °C in minimal selective medium (2% glucose, 0.5% ammonium sulfate, 0.17% yeast nitrogen base, supplemented with appropriate amino acids and nucleotides) or rich medium (2% glucose, 1% yeast extract, 2% bactopeptone).

##### Purification of Recombinant Proteins

Recombinant full-length His_6_-Rrp47 and the C-terminally truncated Rrp47_NT_ protein were purified from *E. coli* lysates, as described previously (35, 38). To purify the Rrp47·Rrp6_NT_ complex, cells expressing either His_6_-Rrp47 or the Rrp6_NT_ domain were lysed together in buffer comprising 50 mm HEPES, pH 7.6, 300 mm NaCl, 10 mm imidazole, 0.5 mm PMSF. The cell extract was clarified by centrifugation at 15,000 × *g* for 30 min, filtered, and then incubated with nickel-nitrilotriacetic acid-agarose beads (Qiagen) for 30 min. The beads were washed with lysis buffer, and retained protein was eluted with lysis buffer containing 250 mm imidazole. The eluate was incubated for 30 min with glutathione-Sepharose beads, washed with lysis buffer, and eluted in lysis buffer containing 25 mm glutathione. The eluate was then concentrated by centrifugation through an Amicon Ultracel (3K) filter and resolved through a 25-ml Superdex 200 column equilibrated with 50 mm HEPES, pH 7.6, 300 mm NaCl using an ÄKTA purification system (GE Healthcare). 0.5-ml fractions were collected and analyzed by SDS-PAGE.

##### Analytical Ultracentrifugation

Peak fractions from the final size exclusion chromatography purification step in 50 mm Hepes, pH 7.6, 500 mm NaCl were concentrated using Amicon Ultracel (3K) filters and analyzed at a concentration of 0.25–1 mg/ml in a Beckman Optima XL-A analytical ultracentrifuge. Velocity sedimentation analyses were performed at 38,000 rpm and at 20 °C. Sedimentation equilibrium analyses were performed at 17,500 rpm, absorption base lines of nonsedimenting material being obtained by overspeeding at 45,000 rpm at the end of the run. Sedimentation velocity and equilibrium profiles were obtained by scanning at a wavelength of 280 nm, using the UV absorption optical system. The extinction coefficient of Rrp47 at 280 nm is estimated to be 8940 m^−1^ cm^−1^, and a partial specific volume ν of recombinant Rrp47 was determined from the amino acid sequence to be 0.733 cm^3^/g (47). Sedimentation coefficients (*s*) and sedimentation coefficient distributions (*c*(*s*) *versus s*) were derived by using the SEDFIT procedure (48). From the rate of spread of the diffusing boundary, an estimate for friction/shape effects is made, and a transformation from a sedimentation coefficient distribution (*c*(*s*) *versus s*) to a molecular weight distribution (*c*(*M*) *versus M*) can be made. Molecular weight estimates (weight averages) were also obtained from sedimentation equilibrium data using the MSTAR procedure (49).

##### Size Exclusion Chromatography

Recombinant full-length Rrp47 and the C-terminally truncated Rrp47_NT_ protein were analyzed by size exclusion chromatography on the ÄKTA purification system, as above. Stokes radius values for the markers bovine γ-globulin (4.8 nm), hen egg ovalbumin (3.05 nm), and horse apomyoglobin (2.12 nm) were taken from the literature (50–52).

##### Protein Cross-linking

Recombinant Rrp47, the Rrp47_NT_ polypeptide, and the Rrp47·Rrp6_NT_ complex (final concentration, ∼40 μm) in 50 mm HEPES, pH 7.6, 300 mm NaCl were incubated with 0.01% glutaraldehyde on ice, and the reactions were quenched by the addition of a half-volume of 1 m Tris-HCl, pH 8. Cross-linked mixtures were resolved through 12% SDS-PAGE gels and analyzed by Western blotting.

##### Protein Analyses

Yeast cell extracts were prepared in lysis buffer comprising 10 mm Tris-HCl, pH 7.6, 150 mm NaCl, 5 mm MgCl_2_, 1 mm PMSF (38). For pull-down experiments, yeast cell extracts were incubated with IgG-Sepharose beads (GE Healthcare), and the retained protein was eluted with 0.5 m acetic acid, as described (1, 32). To minimize protein degradation, extracts for the experiments shown in Figs. 3 and 4*B* were prepared under denaturing conditions by alkaline lysis (53).

For the Rrp47 stability assays, isogenic *GAL*::*rrp47-TAP* and *GAL*::*rrp47-TAP rrp6*Δ strains were grown in 200 ml of minimal medium containing 2% raffinose to an *A*_600_ of 0.5. Rrp47 expression levels were then induced to levels comparable with that observed in a strain expressing an Rrp47-zz fusion from the homologous *RRP47* promoter (32) by the addition of galactose to a final concentration of 2%. Cycloheximide was then added to the cultures to a final concentration of 100 μg/ml, and 20-ml aliquots were harvested at 5-min intervals.

For the protease inhibition experiments, the proteasome inhibitor MG132^4^ (Sigma) or the serine protease inhibitor PMSF was added to *erg6*Δ *rrp47-zz* and *erg6*Δ *rrp47-zz rrp6*Δ mutants to a final concentration of 50 μm or 1 mm, respectively, and cells were harvested at time points after treatment. In control experiments, cells were treated with the same volume of DMSO or isopropyl alcohol carrier solvent.

Western analyses were performed using the following antibodies: peroxidase·anti-peroxidase complex (PAP) (Sigma); mouse anti-Pgk1 (Invitrogen); mouse anti-GFP (Roche Applied Science); mouse penta-His antibody (Qiagen); goat anti-yeast importin-α (Santa Cruz Biotechnology, Inc., Santa Cruz, CA); HRP-conjugated goat anti-mouse secondary antibody (Bio-Rad); and HRP-conjugated mouse anti-goat secondary antibody (Santa Cruz Biotechnology, Inc.). SDS-polyacrylamide gels were stained with colloidal Coomassie Blue solution (InstantBlue, Expedeon). Images were captured and quantified on a G:Box iChemi XL system using the GeneSnap and GeneTools programs (Syngene).

##### Fluorescence Microscopy

Strains expressing Rrp47-GFP or Rrp6-GFP and the isogenic wild-type strain were grown in minimal medium to an *A*_600_ below 1.0. Cells were harvested, stained with an aqueous solution of 1 μg/ml 4′,6-diamidino-2-phenylindole (DAPI), and visualized directly using a Delta Vision RT microscope with a ×100 Olympus objective running SoftWoRx (Applied Precision Instruments). Micrographs of each strain were taken with identical exposure settings using standard FITC and DAPI channels. The background brightness of average intensity projections was equalized, and montages of separate and merged channel images were created using ImageJ software (National Institutes of Health, Bethesda, MD).

##### RNA Analyses

Total RNA was extracted from yeast cell pellets by glass bead extraction in guanidinium isothiocyanate/phenol (54) and further purified using the RNeasy miniprep kit (Qiagen). RNA integrity was confirmed by agarose gel electrophoretic analysis after denaturation with glyoxal. Reverse transcription reactions were performed on 2 μg of DNase I-treated RNA with random hexamer primers, using the Bioline cDNA synthesis kit. Quantitative real-time PCR primers were designed using Primer3Plus software (55), and their specificity was confirmed by melt curve analyses and test PCRs. cDNA reactions were diluted 10-fold, and triplicate quantitative real-time PCRs were performed in a Corbett Rotor-Gene cycler (Qiagen) using the 2× SensiMix SYBR kit (Bioline). Reactions were analyzed using RotorGene 6000 software version 1.7 by the comparative *C_T_* method (56), normalizing *RRP47* mRNA levels against the *SCR1* reference gene. Data from six independent experiments were analyzed.

## RESULTS

### 

#### 

##### Rrp47 Is Expressed as a Homodimer

**FIGURE 1.**
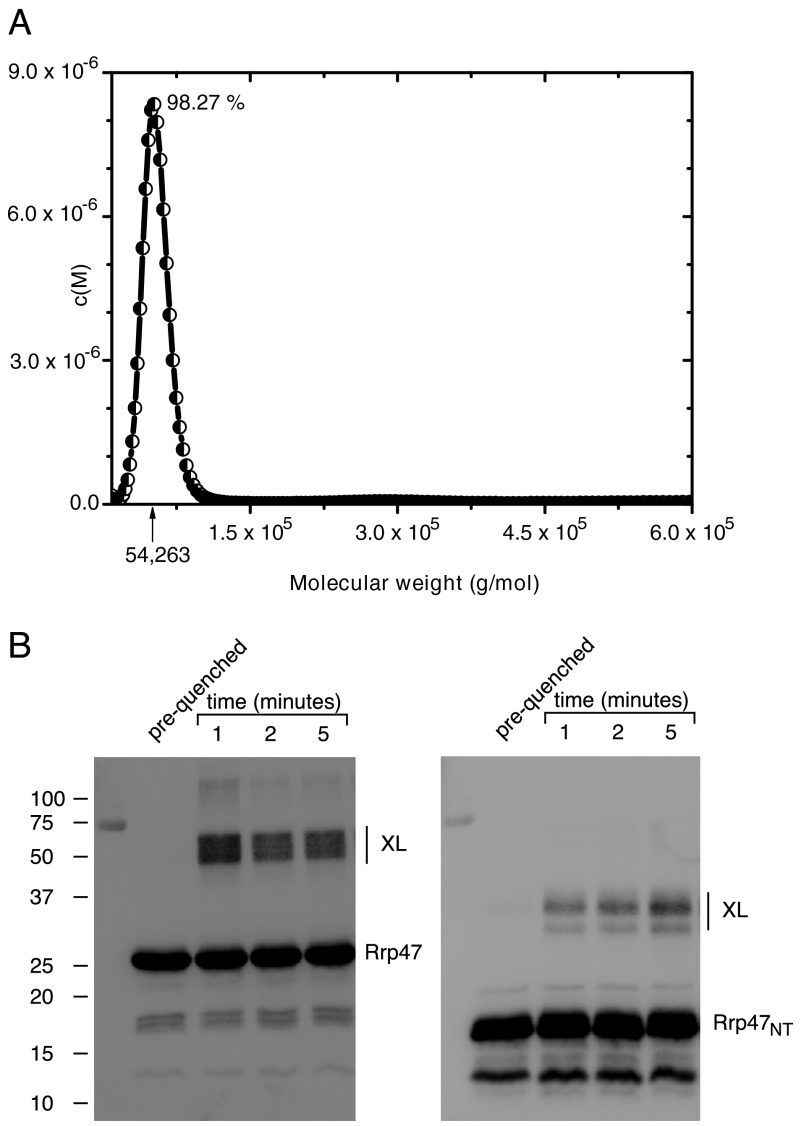
**Recombinant Rrp47 is expressed as a homodimer.** *A*, sedimentation velocity molecular weight distribution analysis of Rrp47. Rrp47 at 0.25 mg/ml (∼10 μm) was centrifuged at 38,000 rpm and at 20.0 °C. The amount of Rrp47 found in the main peak is indicated, along with the corresponding molecular mass of the protein in daltons. *B*, glutaraldehyde cross-linking analysis of full-length Rrp47 and the Rrp47_NT_ domain. Reaction products were resolved by SDS-PAGE and analyzed by Western blotting. The electrophoretic migration of molecular mass markers (sizes in kDa) are given. Cross-linked products (*XL*) are indicated.

To determine the native molecular weight of recombinant Rrp47, we analyzed the purified full-length protein by sedimentation velocity and sedimentation equilibrium analytical ultracentrifugation. The velocity sedimentation profile of His_6_-Rrp47 at 0.25 mg/ml is shown in Fig. 1*A*. A single peak was observed that contained over 98% of the protein, whereas a small fraction was present in larger complexes. After correction for standard solvent conditions, recombinant Rrp47 was deduced to have a sedimentation coefficient *s*_20,_*_w_* of 3.0 S and a molecular mass of ∼54,000 daltons. This value is close to the theoretical value for a homodimer of 50,920 daltons. Similar results were obtained at a higher loading concentration of 1 mg/ml (Table 1). Sedimentation equilibrium experiments were also performed under the same buffer conditions. Concentration distributions at equilibrium, analyzed by the MSTAR routine (49), yielded similar estimates for the weight average molecular weight (Table 1). We conclude that the protein is principally (>98%) dimeric, with a small fraction present in larger aggregates under the stated experimental conditions.

**TABLE 1:** **Summary of the analytical ultracentrifugation results**

Loading concentration	Sedimentation coefficients		Molecular masses	
	*s**^a^*	*s*_20,_*_w_**^b^*	*M**^c^*	*M_w_**^d^*
*mg/ml*	*S*		*Da*	
0.25	2.7 ± 0.1	3.0 ± 0.1	54,000 ± 5000	40,000 ± 5000
0.5				55,000 ± 2000
1.0	2.7 ± 0.1	3.0 ± 0.1	55,000 ± 5000	57,000 ± 5000

*^a^* Measured at 20 °C in the buffer.

*^b^* Corrected to standard conditions of the density and viscosity of water at 20 °C.

*^c^* From sedimentation velocity.

*^d^* From sedimentation equilibrium.

The frictional ratio of a protein correlates with its molecular shape, globular proteins having values around 1.2 and moderately elongated proteins having values up to 2.0 (51). From the calculated *s*_20,_*_w_* value of 3.0 and the theoretical molecular mass of 50,920 daltons, we determined the frictional ratio of recombinant Rrp47 to be ∼1.6. Depending upon the hydration of the protein, this corresponds to an asymmetry ratio (based on a prolate ellipsoid model) of at least 6:1 (57).

The observed elution volume for Rrp47 upon gel filtration corresponded to a Stokes radius of ∼4.5 nm. Using the sedimentation coefficient of 3.0 S and a value for the Stokes radius of 4.5 nm, the molecular mass of the protein is estimated to be ∼57,000 daltons. This value is in good agreement with the molecular weight determined by analytical ultracentrifugation and supports the conclusion that the protein is a non-globular homodimer. The elongated molecular shape of Rrp47 is consistent with the behavior of the protein as an apparent hexamer upon gel filtration analyses (35).

The stoichiometry of the Rrp47 complex was also analyzed in cross-linking experiments. Incubation of full-length Rrp47 protein with glutaraldehyde caused an accumulation of products that exhibited the apparent molecular weight of dimers upon SDS-PAGE analysis (Fig. 1*B*). Approximately 20% of the protein was rapidly converted into cross-linked product. Furthermore, cross-linked dimers were generated upon exposure of Rrp47 to UV light or upon irradiation with visible light in the presence of methylene blue. In addition to the dimer, a low level of higher molecular weight products was observed upon cross-linking with glutaraldehyde (Fig. 1*B*). These larger cross-linked species may reflect the small amount of protein aggregate observed in velocity sedimentation analyses or products arising from transient molecular interactions between dimers in solution. In addition to validating the expression of Rrp47 as a homodimer, cross-linking provided a convenient experimental approach to ascertain changes in the dimeric state of the protein (see below).

We also performed cross-linking reactions and gel filtration analyses on the N-terminal domain of Rrp47 (denoted Rrp47_NT_). This domain is sufficient for the interaction with Rrp6 *in vitro* and functionally complements an *rrp47*Δ mutant when expressed in yeast (38). Incubation of the Rrp47_NT_ domain with glutaraldehyde also led to products the size of dimers but at a considerably reduced yield compared with the full-length protein (Fig. 1*B*). The N-terminal domain of Rrp47 eluted from the gel filtration column as a single peak with an observed Stokes radius of 3.7 nm. Using the theoretical molecular weights for Rrp47 and Rrp47_NT_ dimers and the determined Stokes radii, we estimated from the gel filtration data that Rrp47 and the Rrp47_NT_ domain have frictional ratios of 1.8 and 1.7, respectively. These results are consistent with the analytical ultracentrifugation studies and demonstrate that both the full-length protein and the N-terminal domain of Rrp47 are expressed as non-globular homodimers.

##### Rrp47 Forms a Heterodimer with the Rrp6_NT_ Domain

**FIGURE 2.**
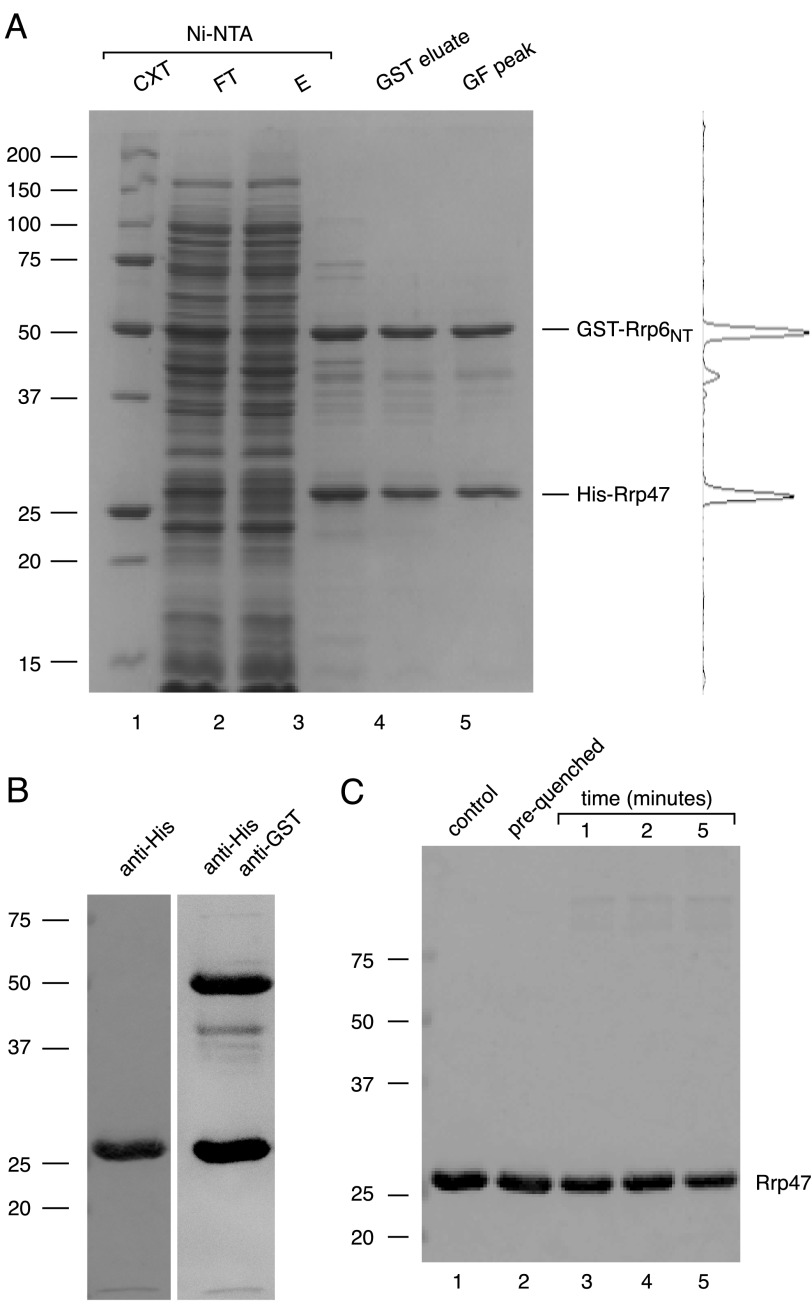
**Rrp47 forms a stoichiometric heterodimer with the Rrp6_NT_ domain.** *A*, Purification of the recombinant Rrp47·Rrp6_NT_ complex. Aliquots of the cell extract (*CXT*; *lane 1*), nickel-nitrilotriacetic acid (*Ni-NTA*) flow-through (*FT*; *lane 2*), and eluate (*E*; *lane 3*) fractions, the eluate upon GST-Sepharose affinity chromatography (*GST eluate*; lane 4), and the peak fraction after gel filtration (*GF peak*; *lane 5*) were resolved by SDS-PAGE, and proteins were visualized by staining with colloidal Coomassie Blue. A densitometric scan of the peak fraction in *lane 5* is shown on the *right. B*, Western analyses of the purified Rrp47·Rrp6_NT_ complex. Images are shown after probing a blot of the GF peak fraction (*A*, *lane 5*) for His-Rrp47 and after reprobing for GST-Rrp6_NT_. *C*, glutaraldehyde cross-linking analysis of the Rrp47·Rrp6_NT_ complex. Reactants were resolved by SDS-PAGE and analyzed by Western blotting using the penta-His antibody. *Lane 1*, non-treated sample. *Lane 2*, prequenched sample. *Lanes 3–5*, samples cross-linked for the times indicated. The positions of molecular mass markers (sizes in kDa) are shown for each *panel*.

Earlier work demonstrated that recombinant Rrp47 interacts with the N-terminal region of Rrp6 (denoted Rrp6_NT_) in pull-down experiments (35, 38). To determine the stoichiometry of Rrp47 bound to Rrp6, the His_6_-Rrp47·GST-Rrp6_NT_ complex was purified from *E. coli* cell lysates by immobilized metal ion chromatography, glutathione affinity chromatography, and size exclusion chromatography. SDS-PAGE analysis of the purified complex revealed two major bands of ∼50 and 25 kDa, corresponding to GST-Rrp6_NT_ and His_6_-Rrp47, that comprised ∼90% of the protein in the peak fractions of the gel filtration profile (Fig. 2*A*). The remaining visible bands on the SDS-polyacrylamide gel arose through proteolytic degradation of the GST-Rrp6_NT_ fusion protein, as revealed by Western analyses using a GST-specific antibody (Fig. 2*B*; see also Ref. 38). Quantification of the electrophoretic profile obtained for the gel filtration peak (Fig. 2*A*), taking into account the abundance and size of the truncated polypeptide bands, provided an estimate of the Rrp6_NT_/Rrp47 ratio to be 0.8:1. Furthermore, the characteristic dimer-sized products observed upon cross-linking of Rrp47 were no longer detected upon Western analyses of the purified Rrp47·Rrp6_NT_ complex after treatment with glutaraldehyde (Fig. 2*C*), upon irradiation with UV light, or upon cross-linking in the presence of methylene blue. The empirically determined Rrp6_NT_/Rrp47 ratio in the purified complex of ∼1:1, together with the lack of detectable dimer product upon cross-linking the complex, support the conclusion that Rrp47 forms a heterodimer upon interaction with the N-terminal domain of Rrp6.

##### Rrp6 Interaction Is Required for Rrp47 Stability in Yeast

**FIGURE 3.**
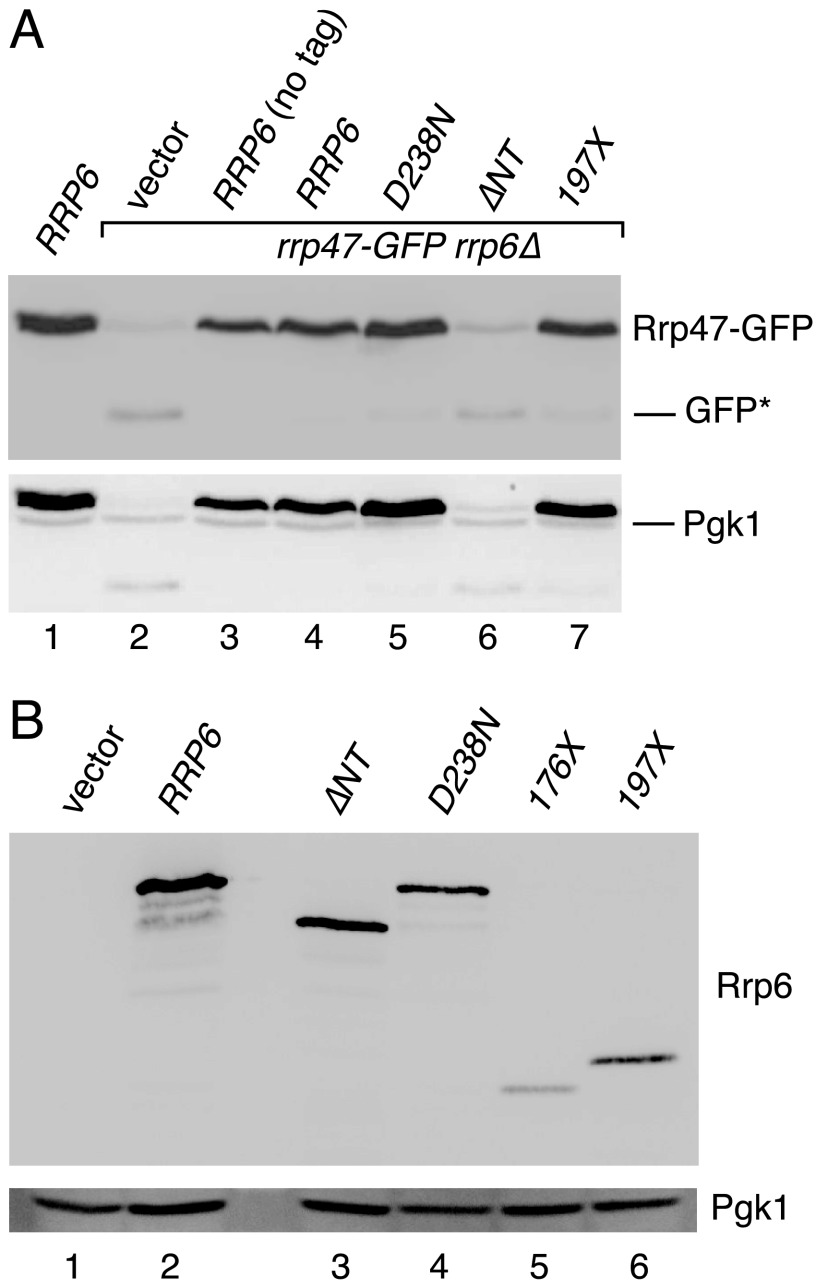
**Rrp47-GFP depletion in *rrp6* mutants correlates with accumulation of a degradation intermediate.** *A*, Western analysis of Rrp47-GFP in *rrp6* mutants. Extracts from a wild-type *RRP6* strain (*lane 1*) and an isogenic *rrp47-GFP rrp6*Δ strain transformed with either the control vector (*lane 2*), a plasmid encoding non-tagged Rrp6 (*lane 3*), or plasmids encoding zz epitope-tagged Rrp6 fusions (*lanes 4–7*) were analyzed. The D238N mutant is a full-length, catalytically inactive protein. The ΔNT fusion lacks the first 211 residues of Rrp6. The 197X mutant encodes the first 196 residues of Rrp6. The Western blot was initially probed for Rrp47-GFP (*top*) and subsequently reprobed with anti-Pgk1 antibody (*bottom*). *B*, Western analysis of the relative expression levels of zz-Rrp6 proteins. Extracts were analyzed from an *rrp47-GFP rrp6*Δ strain transformed with the control vector (*lane 1*) and zz-Rrp6 fusions (*lanes 2–6*), as indicated. Blots were first probed with the Pgk1 antibody (*bottom*) and then with the PAP antibody (*top*) to detect the zz fusion proteins.

We have previously reported that the expression level of Rrp47 is significantly reduced in yeast strains expressing a mutant Rrp6 protein lacking the N-terminal domain (35). This earlier study employed strains expressing Rrp6 and Rrp47 fusion proteins containing the same epitope tag and used the *GAL* promoter to drive high expression levels of the Rrp6_NT_ domain. To readdress the requirements within Rrp6 for Rrp47 expression, Western analyses were performed on cell lysates of *rrp47-GFP rrp6*Δ strains harboring plasmids that encode one of a number of different zz-tagged Rrp6 proteins (Fig. 3*A*). The Rrp6 proteins analyzed included a full-length Rrp6, the catalytically inactive D238N mutant (17), a deletion mutant lacking the N-terminal domain (ΔNT), and a truncation mutant expressing only the N-terminal domain (197X); a wild-type *RRP6* strain and an *rrp6*Δ mutant transformed with the vector were also analyzed as positive and negative controls for normal Rrp47 expression (Fig. 3*A*, *lanes 1* and *2*, respectively). These experiments confirmed that expression of either the full-length Rrp6 or only the N-terminal domain was sufficient to allow recovery of Rrp47 expression levels, whereas Rrp47 was depleted in the ΔNT mutant to a level similar to that observed in the *rrp6*Δ mutant (Fig. 3*A*). The expression level of Rrp47 in the catalytically inactive D238N mutant was comparable with that observed in the wild-type strain. Western analyses revealed all tested Rrp6 proteins to be stably expressed, whereas a shorter N-terminal domain construct terminating at residue Pro-176 gave lower expression levels (Fig. 3*B*).

Moreover, the decrease in Rrp47-GFP fusion protein seen in the *rrp6*Δ and ΔNT mutants was associated with the accumulation of a ∼25-kDa polypeptide that was detectable with the anti-GFP antibody (labeled GFP* in Fig. 3*A*, *lanes 2* and *6*) and that is comparable in size with the GFP domain. Initial Western analyses on extracts prepared under native conditions revealed little or no full-length Rrp47-GFP protein in the *rrp6*Δ mutant, whereas the GFP* band was clearly detected. The GFP domain is known to be particularly resistant to proteolytic degradation *in vivo* and has a half-life of ∼7 h in yeast (58, 59), suggesting that the GFP* fragment results from proteolysis of the Rrp47-GFP fusion protein. Comparison of different extraction procedures revealed that the minimal degree of Rrp47-GFP degradation was observed in extracts prepared by cold alkaline lysis (53). The Western blot data shown in Fig. 3 were obtained using this technique. Quantitative Western analyses of cell extracts obtained by cold alkaline lysis showed that the steady state level of the GFP* band is ∼1.8-fold greater than that of the full-length protein (*n* = 6; S.E. = 0.15).

The correlation between normal expression levels of Rrp47 and the availability of the N-terminal domain of Rrp6 with which it directly interacts, together with the accumulation of a polypeptide the size of the GFP domain in *rrp47-GFP* strains not expressing the Rrp6_NT_ domain, strongly suggest that Rrp47 is stabilized against proteolysis upon interaction with Rrp6.

**FIGURE 4.**
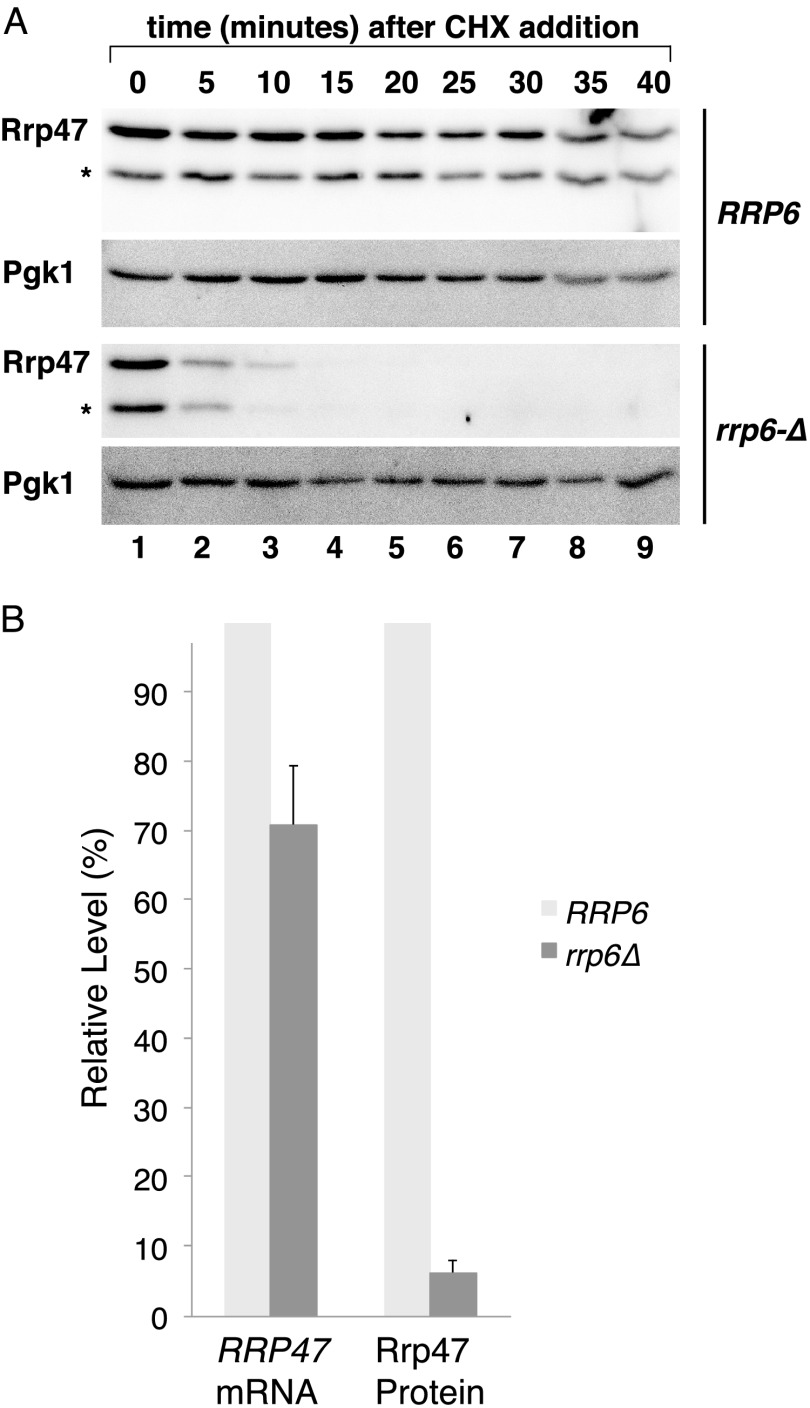
**Rrp47 protein is unstable in the absence of Rrp6.** *A*, Rrp47 stability assays in an isogenic wild-type, *RRP6* strain and an *rrp6*Δ mutant bearing a *GAL*-regulated Rrp47-zz fusion protein. The strains were pregrown in raffinose-based medium, and Rrp47 expression was then induced to normal levels by the addition of galactose. New protein synthesis was subsequently blocked by the addition of cycloheximide and the reduction of Rrp47 levels followed by Western analyses. The lower band, indicated with an *asterisk*, is a proteolytic Rrp47-zz degradation product. The Western blots were also probed for Pgk1. *B*, relative expression levels of *RRP47* mRNA and protein observed in a wild-type *RRP6* strain and an isogenic *rrp6*Δ mutant strain. *RRP47* mRNA levels were determined by quantitative RT-PCR and standardized to *SCR1* levels. Western analyses were performed on alkaline-lysed cells and standardized to Pgk1 levels. *Error bars*, positive range of the S.E.

**FIGURE 5.**
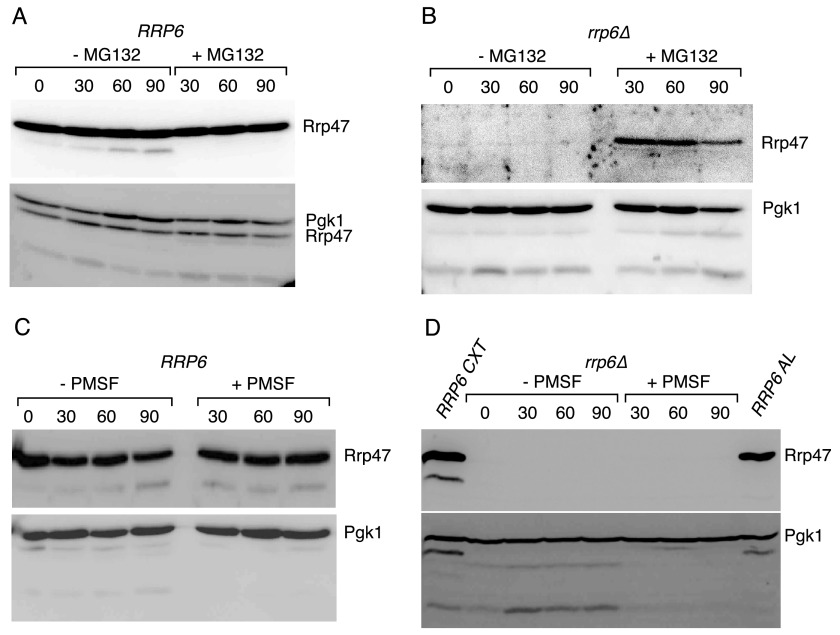
**Rrp47 levels in *rrp6*Δ mutants increase upon treatment with MG132.** Western analyses of Rrp47 in isogenic *RRP6* and *rrp6*Δ strains upon treatment with either MG132, PMSF, or vehicle solvent. Cells were harvested before treatment and 30, 60, and 90 min thereafter. Cell extracts were resolved by SDS-PAGE and analyzed by Western blotting, using the PAP antibody to detect the Rrp47-zz fusion protein and using an anti-Pgk1 antibody as a loading control. *A*, wild-type *RRP6* strain treated with MG132. *B*, *rrp6*Δ mutant treated with MG132. *C*, *RRP6* strain treated with PMSF. *D*, *rrp6*Δ mutant treated with PMSF. The sample to the *left* in *D* labeled *RRP6 CXT* is a native cell extract from an *RRP6* strain. The sample to the *right* in *D* labeled *RRP6 AL* is an alkaline lysate from an *RRP6* strain.

To determine whether Rrp47 is degraded more rapidly in an *rrp6*Δ mutant than in an *RRP6* wild-type strain, we analyzed the stability of the protein in translational shut-off experiments. The decrease of a newly synthesized pool of an epitope-tagged Rrp47-TAP fusion protein in a wild-type *RRP6* strain and an *rrp6*Δ mutant was followed by Western analysis of cell lysates after inhibition of further protein synthesis with the translational inhibitor cycloheximide. Expression of the fusion protein under the control of the *GAL* promoter ensured tight induction of protein expression and allowed normal Rrp47 levels to be attained in the *rrp6*Δ mutant. Newly synthesized Rrp47 was rapidly depleted in the *rrp6*Δ mutant, with little detectable signal after 15 min (Fig. 4*A*). In contrast, the level of newly synthesized Rrp47 in wild-type *RRP6* cells remained relatively constant throughout the duration of the 40-min time course following translation inhibition. The analyses shown in Fig. 4*A* were performed on native cell extracts. Under these conditions, a smaller polypeptide fragment is observed (denoted with an *asterisk*) that is absent when the cells are lysed under denaturing conditions (see Fig. 5*D*, *bottom*). Furthermore, we determined the relative steady state levels of Rrp47 protein (in denatured cell extracts) and *RRP47* mRNA transcripts observed in a wild-type *RRP6* strain and an isogenic *rrp6*Δ mutant by Western analyses and quantitative RT-PCR. Rrp47 protein levels in the *rrp6*Δ mutant were reduced to ∼15-fold less than that seen in the wild-type *RRP6* strain (6.1%, S.E. = 2.1%, *n* = 5), whereas *RRP47* mRNA levels were only slightly decreased (70.7%, S.E. = 8.6%, *n* = 6) (Fig. 4*B*). The above results are consistent with newly synthesized Rrp47 undergoing one of two fates; either the protein is incorporated into a heterodimer with Rrp6, or it is targeted to rapid proteolytic degradation.

##### Rrp47 Is Degraded by the Proteasome in the Absence of Rrp6

To address the mechanism by which Rrp47 is rapidly degraded in *rrp6*Δ cells, wild-type *RRP6* and *rrp6*Δ strains expressing the Rrp47-zz epitope-tagged fusion protein (32) were treated with either the proteasome inhibitor MG132 or the serine protease inhibitor PMSF (60), which targets the vacuolar proteinase A (Pep4 in yeast). The addition of MG132 to cultured *rrp6*Δ cells caused Rrp47 to accumulate considerably above the level observed in the untreated cells (Fig. 5*B*). Treatment of wild-type cells with MG132 had no noticeable effect on the levels of full-length Rrp47. In contrast, treatment with PMSF did not lead to a recovery of Rrp47 expression in the *rrp6*Δ mutant (Fig. 5*D*). Truncated polypeptides of Pgk1, a known substrate of the vacuolar autophagy pathway (59), could be detected in MG132-treated and in control cells (Fig. 5, *A* and *B*), but their levels were depleted in cells treated with PMSF (Fig. 5, *C* and *D*). Consistent with these results, depleted Rrp47 levels in the *rrp6*Δ mutant were not increased in strains that also lacked Pep4. We conclude that Rrp47 degradation proceeds via a proteasome-mediated pathway.

An N-terminally tagged Rrp6 fusion protein could be readily overexpressed in yeast using a high copy number plasmid. Notably, overexpression of Rrp6 did not lead to an increase in the expression level observed for Rrp47. These data suggest that Rrp47 levels are not limited solely by the availability of Rrp6 and imply that additional mechanisms exist to control the expression of the *RRP47* gene.

##### Rrp47 and Rrp6 Are Independently Localized to the Nucleus

**FIGURE 6.**
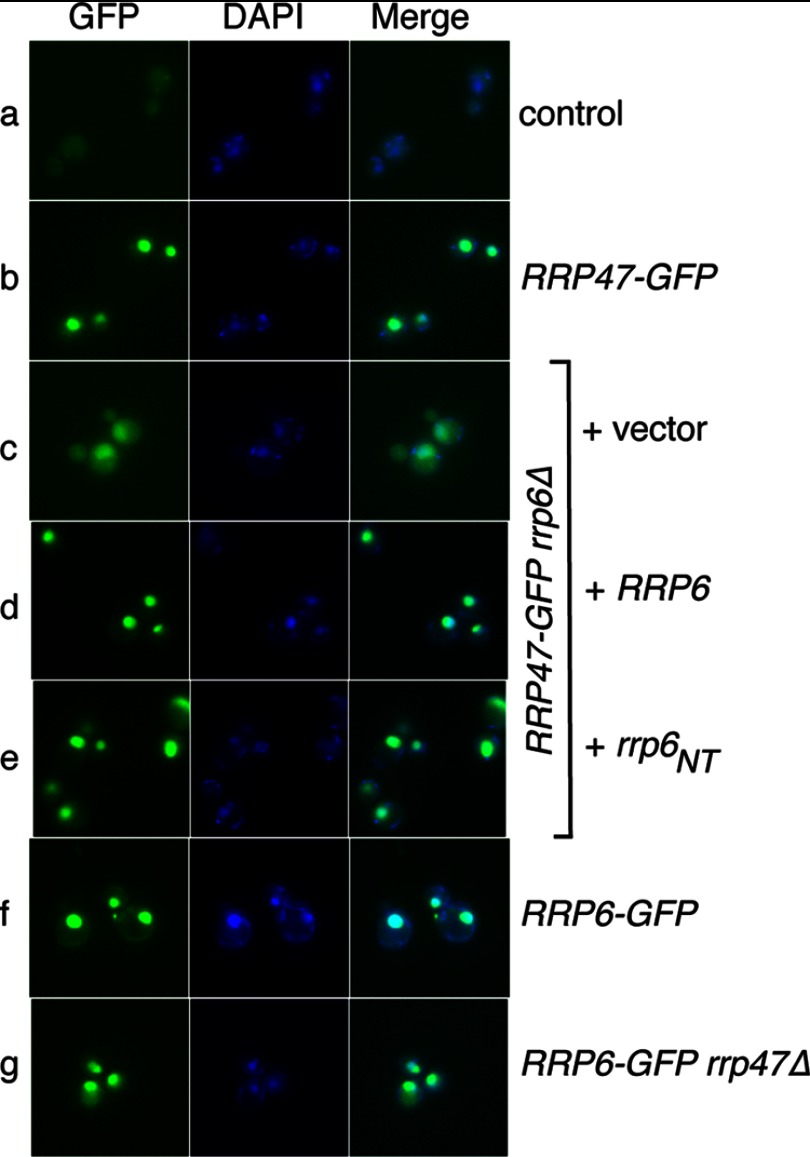
**Nuclear localization of Rrp47 and Rrp6 is independent.** Shown is localization of Rrp47 and Rrp6 GFP fusion proteins in strains either expressing the other protein or bearing mutant alleles of the corresponding gene. GFP, DAPI, and merged images are shown for representative cells of strains expressing no GFP fusion (*a*), Rrp47-GFP (*b*), Rrp47-GFP and lacking Rrp6 (*c*), Rrp47-GFP and full-length Rrp6 (*d*), Rrp47-GFP and the Rrp6_NT_ domain (*e*), Rrp6-GFP (*f*), and Rrp6-GFP and lacking Rrp47 (*g*).

To address whether Rrp47 and Rrp6 are localized to the nucleus independently of one another, we compared the subcellular localization of Rrp47-GFP and Rrp6-GFP fusion proteins expressed from their homologous promoters in non-fixed, wild-type cells with that observed in the respective *rrp6*Δ or *rrp47*Δ mutants. Consistent with previous findings (45), Rrp47-GFP gave a clear nuclear signal in wild-type *RRP6* cells (Fig. 6*b*). Nuclear foci of Rrp47-GFP were also clearly visible in the *rrp6*Δ mutant (Fig. 6*c*). As expected from the Western analyses of Rrp47 expression levels (Fig. 3), the intensity of the signal detected from the Rrp47-GFP protein in the *rrp6*Δ mutant was considerably lower than in the *RRP6* wild-type strain. Quantification of the GFP signal showed that the cytoplasmic fluorescence observed in the Rrp47-GFP *rrp6*Δ cells (Fig. 6*c*) was not more than that observed in the Rrp47-GFP wild-type strain (data not shown).

Western analyses of alkaline-lysed cells revealed that the majority of the Rrp47-GFP fusion protein observed in the *rrp6*Δ mutant exists as a stable degradation intermediate with a molecular weight the size of the GFP domain (Fig. 3). Expression of the GFP domain alone does not result in nuclear foci in yeast (61), and passive import of GFP-sized molecules across the nuclear pore is impeded ∼10-fold in permeabilized HeLa cells (62). Therefore, proteolytic release of the GFP domain from the Rrp47-GFP fusion protein in the cytoplasm would not lead to its accumulation within the nucleus. These data show that the Rrp47-GFP fusion protein can be imported into the nucleus independently of Rrp6. Because the released GFP domain represents a marker for the proteolysis of Rrp47-GFP, it follows that degradation of Rrp47-GFP in the *rrp6*Δ mutant most probably occurs within the nucleus.

Expression of full-length Rrp6 complemented the reduced levels of Rrp47-GFP observed in the *rrp6*Δ mutant and led to the recovery of a bright fluorescence signal in the nucleus (Fig. 6*d*). A similar effect was observed when only the Rrp6_NT_ domain was expressed (Fig. 6*e*). Thus, both full-length Rrp6 and the Rrp6_NT_ domain allow nuclear accumulation of Rrp47 as well as recovery of normal expression levels of the protein (Fig. 3). Because the N-terminal region of Rrp6 is sufficient to bind Rrp47 (35) and to allow stable expression in yeast (Fig. 3), we infer that the signal observed in Fig. 6*e* reflects the assembled Rrp47·Rrp6_NT_ complex.

As previously reported (2, 17), we detected a strong nuclear localization for Rrp6 (Fig. 6*f*). Rrp6-GFP also accumulated in the nucleus in the *rrp47*Δ mutant (Fig. 6*g*). We conclude that Rrp6 and Rrp47 can be imported into the nucleus independently of each other.

##### Rrp6, but Not Rrp47, Is Associated with Srp1

**FIGURE 7.**
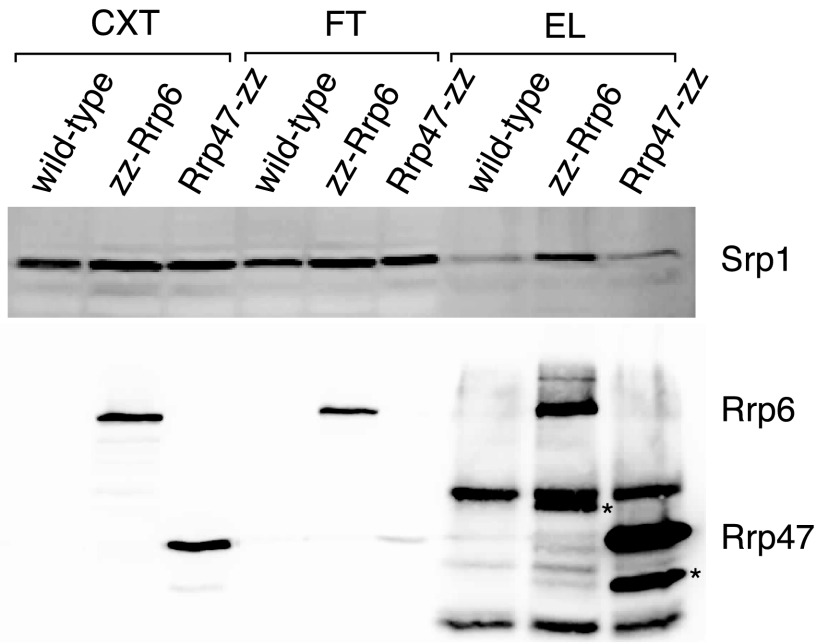
**Rrp47 is not associated with the Rrp6·Srp1 complex.** Western blot analysis of pull-downs on extracts from yeast strains expressing epitope-tagged zz fusions of Rrp6 or Rrp47, using an Srp1-specific antibody. A non-tagged, wild-type strain was used as a negative control. Cell extracts (*CXT*) and the flow-through (*FT*) and eluate (*EL*) fractions obtained upon fractionation using IgG-Sepharose beads were resolved through 12% SDS-polyacrylamide gels and analyzed by Western blotting. The *top panel* shows a Western blot using an Srp1-specific antibody. The *bottom panel* shows a Western blot using the PAP antibody. Rrp6 and Rrp47 proteolytic fragments are denoted with *asterisks*.

TAP-tagged protein purification experiments using Rrp6 as a bait have revealed a stable association with the Srp1·Kap95 importin α/β complex (33, 43) in addition to interactions with the exosome core complex. In contrast, analogous experiments using Rrp47 as the bait did not detect an association with the importin complex (33). To address this further, we directly compared pull-downs of Rrp6 and Rrp47 proteins for the presence of associated Srp1 (yeast importin-α) by Western analyses (Fig. 7). Srp1 was reproducibly enriched in the pull-down using epitope-tagged Rrp6, compared with the non-tagged wild-type control strain. In contrast, the levels of Srp1 associated with Rrp47 were close to the observed background. Notably, Rrp47 was more efficiently depleted from the cell extract than Rrp6. A number of additional bands are visible in the eluate fractions that do not correspond to either full-length Rrp6 or Rrp47. The nonspecific bands observed in all eluate fractions upon incubation with the PAP antibody result from interaction with traces of IgG heavy and light chains, whereas proteolytic fragments of Rrp6 and Rrp47 are also detected in the enriched eluates (denoted by *asterisks* in Fig. 7). These results suggest that a fraction of Rrp6 in cell extracts is stably associated with Srp1 but not Rrp47 and that there is not a comparable stable complex containing Rrp47 and Srp1. The Srp1-associated fraction of Rrp6 presumably represents a complex that is primed for translocation across the membrane or release of its cargo within the nucleus. These results are consistent with the existence of independent nuclear import routes for Rrp6 and Rrp47 prior to assembly of the Rrp6·Rrp47 heterodimer within the nucleus.

## DISCUSSION

The activity of Rrp6 in RNA processing and discard pathways is strongly influenced by its interaction with Rrp47, but the precise basis of this effect is presently not understood. Here we have addressed the assembly of the yeast Rrp6·Rrp47 complex. Our data reveal that the Rrp6·Rrp47 complex is formed after mutually independent import of each protein into the nucleus and that Rrp47 is rapidly degraded if Rrp6 is not available for interaction. Rrp47 is expressed as a homodimer that is disassembled upon interaction with Rrp6, this structural rearrangement presumably underlying its ability to avoid degradation upon Rrp6 binding. Nuclear assembly of the Rrp6·Rrp47 complex would ensure that RNA degradation-competent Rrp6·Rrp47 complexes are generated only within the nuclear compartment where they function, whereas degradation of Rrp47 in the absence of Rrp6 would prevent its potentially deleterious independent expression.

The homodimeric nature of Rrp47 was revealed by hydrodynamic analyses and further supported by chemical and photochemical cross-linking experiments. The frictional ratio determined for Rrp47 of ∼1.6 is indicative of an extended, non-globular protein. For comparison, a fragment of the protein tenascin with a molecular weight and frictional coefficient similar to those of Rrp47 comprises an extended chain of five fibronectin type III domains (51, 64). Values for the frictional coefficient of Rrp47 obtained by sedimentational analyses (1.6) and size exclusion chromatography (1.8) both suggest an extended protein shape (51). Moreover, the gel filtration analyses reveal that full-length Rrp47 and the N-terminal region of the protein are comparably elongated. Thus, the non-globular shape of Rrp47 is not simply due to the presence of an extended, unstructured C-terminal region.

Rrp6 has been shown to interact with Rrp47 by affinity capture analyses of protein complexes in yeast (32, 43, 65–68) and by *in vitro* reconstitution studies (35, 38). However, the stoichiometry of this complex has not been previously addressed. SDS-PAGE analysis of the purified Rrp47·Rrp6_NT_ complex suggested a 1:1 stoichiometry between the constituent proteins. This implies that the Rrp47 homodimer must initially dissociate to enable formation of the Rrp6·Rrp47 heterodimer. Dissociation of Rrp47 upon interaction with Rrp6 is supported by the observations that the intermolecular cross-links readily detected upon analysis of the homodimer were no longer observed when the reaction was performed using the purified Rrp47·Rrp6_NT_ complex as a substrate. Our cross-linking data reveal that the Rrp47 homodimer interface lies within the first 120 amino acid residues, which closely maps to the Sas10/C1D domain that is minimally required for interaction with Rrp6 (38). It remains to be determined whether the Rrp6_NT_·Rrp47 heterodimer also has an extended, non-globular structure.

Western analyses of Rrp47 levels after translational arrest revealed that Rrp47 is rapidly degraded in the *rrp6*Δ mutant, and steady state analyses demonstrated that expression of the N-terminal region of Rrp6 was required for normal expression of Rrp47. This strongly suggests that formation of the heterodimer with Rrp6 enables Rrp47 to evade proteolytic degradation. The availability of Rrp6 may provide a simple regulatory mechanism to ensure appropriate expression levels of Rrp47. Thus, fluctuations in the expression level of Rrp6, such as the depletion observed during meiosis (10), may be sufficient to ensure that Rrp47 expression patterns follow a similar profile. Furthermore, proteolysis of Rrp47 may prevent potentially deleterious effects of Rrp47 expression in the absence of Rrp6. It is highly feasible that the expression of a nucleic acid-binding protein in the absence of its associated nuclease may titrate out RNAs and block their efficient degradation or processing by alternative factors. Rrp47 is expressed in yeast at levels close to its *K_d_* for RNA or DNA (37), so an increased expression would be predicted to cause a sharp increase in nucleic acid binding. Protein capture experiments have revealed that Rrp47 can also interact with other proteins associated with RNA processing or degradation in an RNA-independent manner (38), thereby potentially leading to their depletion from active complexes in the absence of Rrp6. In this vein, overexpression of C1D in human tumor cell lines has been shown to cause an increased incidence of apoptosis (69). In a more general sense, *rrp6*Δ mutants have been widely analyzed to deduce the role of the exosome complex in nuclear RNA processing and surveillance events; the interpretation of data obtained from studying cells lacking Rrp6 should also take into account documented secondary effects, such as the depletion of Rrp47 and the observed decrease in activity of the other exosome-associated nuclease, Rrp44 (70).

**FIGURE 8.**
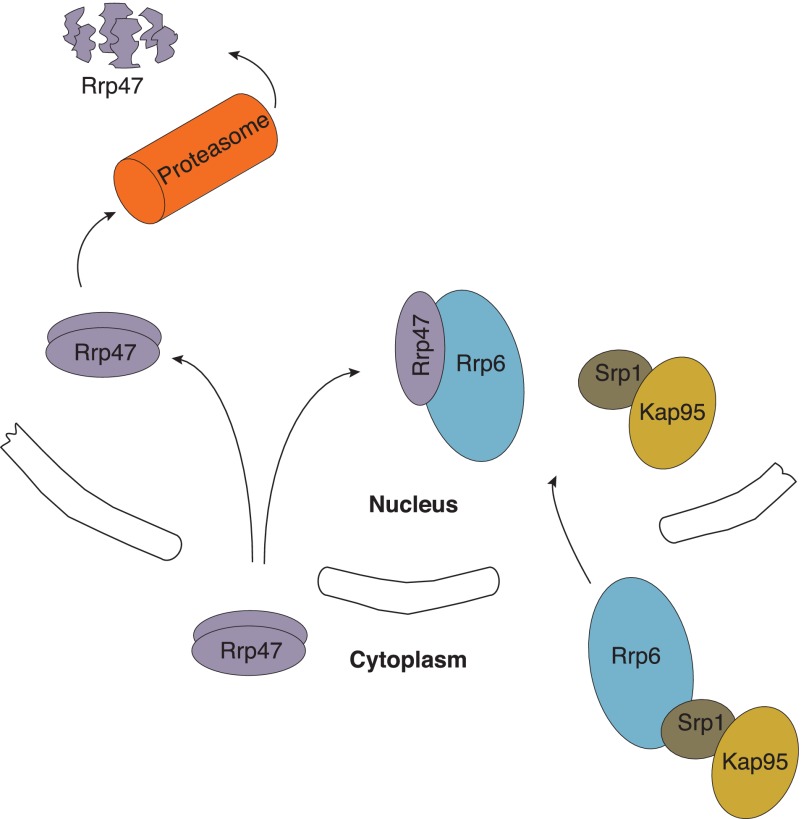
**Model for assembly of the Rrp6·Rrp47 complex.** The Rrp47 homodimer is imported independently of Rrp6, the latter protein being translocated across the nuclear membrane by the importin heterodimer Srp1·Kap95. The Rrp6·Rrp47 heterodimer is assembled in the nucleus, generating the RNA-processing/degradation-competent ribonuclease complex. In the absence of Rrp6, the Rrp47 homodimer is degraded by the nuclear proteasome.

Our studies show that Rrp47 is degraded in cells lacking Rrp6 by a vacuole-independent, proteasome-mediated pathway. At steady state, Rrp47 lacks the N-terminal methionyl residue but is not *N*-acetylated (71). Removal of the N-terminal methionine of Rrp47 generates an N-terminal glutamate residue that could potentially act as a substrate for the cytoplasmic arginyl-tRNA-protein transferase Ate1 in the absence of Rrp6 binding and target the protein to the Arg/N-end rule pathway (72). However, genetic depletion of the Arg/N-end rule pathway factors Ate1 or Ubr1 did not lead to a significant increase in the levels of Rrp47 observed in the *rrp6*Δ mutant.^5^ In contrast, our data support a model wherein assembly of the Rrp6·Rrp47 complex or Rrp47 degradation occurs in the nucleus (Fig. 8). First, Rrp47-GFP and Rrp6-GFP fusion proteins were both localized to the nucleus independently of one another. Furthermore, the Rrp47-GFP fusion protein observed to be localized to the nucleus in the *rrp6*Δ mutant was detected predominantly as a trapped degradation intermediate with the size of the GFP domain; because expression of GFP alone does not lead to its accumulation in the nucleus, this domain could not be initially released by proteolysis in the cytoplasm and then subsequently localized to the nucleus. Finally, deletion of a canonical bipartite nuclear localization signal in the C-terminal region of Rrp6 causes its mislocalization to the cytoplasm (22, 25), and pull-down experiments revealed an association between Rrp6 and the importin-α subunit Srp1. In contrast, observed Srp1 levels bound to Rrp47 were not above background values. We envisage that this assembly pathway enables activated Rrp6 complexes to be confined to the nucleus and provides the basis for an autoregulatory mechanism to block the expression of Rrp47 in the absence of Rrp6.

The proposed model supposes that Rrp6 and Rrp47 interact with one another after they have been imported into the nucleus. At present, we are only able to speculate about the mechanism(s) that may prevent these two proteins from interacting prematurely in the cytoplasm. Maintaining the two proteins separate before import could be coupled to the use of two independent import mechanisms. Thus, interaction of either Rrp6 or Rrp47 with their cognate importin may prevent the formation of the Rrp6·Rrp47 complex. Importin binding would provide the most elegant solution to avoid premature assembly of the Rrp6·Rrp47 complex because the interaction surface would become available only after import into the nucleus and release of the importin. Some importins are thought to also function as chaperone proteins for highly basic RNA-binding proteins, such as Rrp47 (73). Other chaperone proteins may function to prevent Rrp6 from interacting with Rrp47 in the cytoplasm. Furthermore, nuclear translocation is often regulated by protein phosphorylation. Rrp6 can be phosphorylated at multiple sites (63), but the functional relevance of these modifications is unclear; although none of these modifications occur within the PMC2NT domain, it is nevertheless feasible that one or more modifications might suppress the ability of Rrp6 to interact with Rrp47. More detailed future studies on the Rrp6-independent nuclear import of Rrp47 will be made feasible by the isolation of *rrp47* mutants that are stable in the absence of Rrp6.

## Footnotes

5 M. Feigenbutz and P. Mitchell, unpublished data.

## Footnotes

4 The abbreviations used are:

MG132: carbobenzoxyl-Leu-Leu-leucinal
zz: epitope tag consisting of two copies of the z domain of protein A from *S. aureus*
PAP: peroxidase·anti-peroxidase complex.
